# Influence of H7N9 virus infection and associated treatment on human gut microbiota

**DOI:** 10.1038/srep14771

**Published:** 2015-10-22

**Authors:** Nan Qin, Beiwen Zheng, Jian Yao, Lihua Guo, Jian Zuo, Lingjiao Wu, Jiawei Zhou, Lin Liu, Jing Guo, Shujun Ni, Ang Li, Yixin Zhu, Weifeng Liang, Yonghong Xiao, S. Dusko Ehrlich, Lanjuan Li

**Affiliations:** 1 State Key Laboratory for Diagnosis and Treatment of Infectious Diseases, the First Affiliated College of Medicine, Zhejiang University, 310003 Hangzhou, China; 2 Collaborative Innovation Center for Diagnosis and Treatment of Infectious Diseases, Zhejiang University, 310003 Hangzhou, China; 3 Metagenopolis, Institut National de la Recherche Agronomique, 78350, Jouy en Josas, France; 4 King’s College London, Centre for Host-Microbiome Interactions, Dental Institute Central Office, Guy’s Hospital, London Bridge, London SE1 9RT, UK

## Abstract

Between March and June, 2013, forty H7N9 patients were hospitalized in our hospital. Next-generation sequencing technologies have been used to sequence the fecal DNA samples of the patient, the within sample diversity analysis, enterotyping, functional gene and metagenomic species analysis have been carried on both the patients and healthy controls. The influence of associated treatment in H7N9 infected patients is dramatic and was firstly revealed in species level due to deep sequencing technology. We found that most of the MetaGenomic Species (MGS) enriched in the control samples were *Roseburia inulinivorans* DSM 16841, butyrate producing bacterium SS3/4 and most of MGS enriched in the H7N9 patients were *Clostridium* sp. 7 2 43FAA and *Enterococcus faecium.* It was concluded that H7N9 viral infection and antibiotic administration have a significant effect on the microbiota community with decreased diversity and overgrowth of the bacteria such as *Escherichia coli* and *Enterococcus faecium*. Enterotype analysis showed that the communities were unstable. Treatment including antivirals, probiotics and antibiotics helps to improve the microbiota diversity and the abundance of beneficial bacteria in the gut.

In the spring of 2013, a novel avian-origin influenza virus (H7N9) rings alarm bells in China^1^,^2^,^3^. The H7N9 virus originated from multiple reassortment events that had not been identified previously in human populations. The hem agglutinin gene (H7) of H7N9 might have evolved from avian influenza viruses of duck origin, whereas the neuraminidase gene (N9) was identical to H7N9 isolates from Korean ducks and wild birds, and the internal genes probably originated from an earlier H9N2 lineage^4^. We previously reported that human infections with H7N9 had fever and rapidly progressive pneumonia that did not respond to antibiotics^1^. However, acute respiratory distress syndrome (ARDS), septic shock and multi-organ failure were noted in some fatal cases^2^,^5^.

Between March 7 and June 28th, 2013, a total of 40 human cases of infection with H7N9 influenza were hospitalized in the First Affiliated Hospital, College of Medicine, Zhejiang University. No human infections with H7N9 viruses have been reported until this outbreak, at this time, there is little clinical experience with the treatment of H7N9 infection. To improve therapeutic outcomes and reducing mortality, a treatment regimen combining antiviral therapy, anti-shock therapy, anti-ARDS therapy, anti-infection therapy, maintaining the balance of intestinal microbiota and water-electrolyte was conducted in our hospital. Of the patients who were hospitalized, 20% (8/40) died. By contrast, the overall proportion of fatal cases in other provinces was 39.4% (37/94 excluding Zhejiang patients, as of August 31th, 2013).

The indigenous microbiota plays a pivotal role for the prevention and treatment of microbial infections, and it is sometimes referred to as a “forgotten organ”^6^,^7^. Since next-generation sequencing technologies emerged, much work has been done in gut microbiota field and greatly accelerated the research in this field. The Human Microbiome Project (HMP) founded by NIH has sequencing microbiota from different anatomical sites among 242 healthy individuals and generated the largest human microbiome gene resource so far^8^. Metagenomcis of Human Intestinal Tract (MetaHIT) founded by European commission has generated the first human gut gene catalogue and identified enterotypes which are independent of geographic origin in the human gut microbiota^9^. Another gut microbiota study from 345 Chinese individuals with type-2 diabetes (T2D) found 60,000 T2D-associated genes and reported a combination of the genes that could be used to accurately diagnose the disease^10^.

Remarkably, chronic complex diseases have been associated with gut microbiota. Although the intestinal microbiota is generally stable in healthy individuals over long periods of time, antibiotics can significantly reshape the gut microbiota, allowing exogenous microbes to outgrow commensal bacteria and cause permanent changes in varying states of disease^6^,^11^,^12^. Probiotic agents, which beneficially affect the host by improving the gut microbial balance, have been used for the prevention and treatment of respiratory tract diseases to avoid bacterial translocation^13^,^14^. It is noteworthy that gastrointestinal distress symptoms were observed in some of our recently reported cases of H7N9 infection^1^,^5^. Additionally, most of patients received antibiotic therapy within six hours after admission and also received probiotic agents in our hospital. Influenza virus infection and subsequent therapies may affect human microbiota in a model system^15^,^16^, however, the consequences of H7N9 infection in humans and the interplay between viral pathogens and the microbiota of the host remains unknown.

In this study, we describe the influence of antibiotics and probiotics treatment on H7N9 patients’ gut microbiota, and show that viral infection and associated antibiotic and probiotics usage have a significant effect on the microbiota.

## Results

A total of 26 patients were enrolled, we were not able to collect the fecal samples from the other 14 patients due to different condition of each patient. In total, 93 stool samples were taken from 26 patients (Table S1); the first sample was taken on the day of admission. The median age of patients is 57 years (range, 30 to 80). Most of the patients were elderly, with 42.3% aged older than 65 years, 5 patients were over 75 years of age (19.2%). Males predominate in number over females, about 69.2% were men. Of the 26 patients, 24 were discharged from hospital and 2 died. This study represents 65% (26/40) of all lab confirmed cases in our hospital. 17 patients (65.4%) received antibiotic therapy within six hours after admission. Commonly used antibiotics included piperacillin-tazobactam (n = 12), moxifloxacin (n = 9), sulbactam-cefoperazone (n = 3), imipenem-cilastatin (n = 4), vancomycin (n = 7), and piperacillin-sulbactam (n = 3). Remarkably, 24 patients (92.3%) were treated with *Clostridium* tablets, 2 patients (7.7%) received *B. Subtilis* and *E. faecium* enteric coated capsules and *Clostridium* tablets, and 1 patient received *Clostridium* tablets and *Bacillus* capsules (Tables 1 and S1).

**Figure 1:**
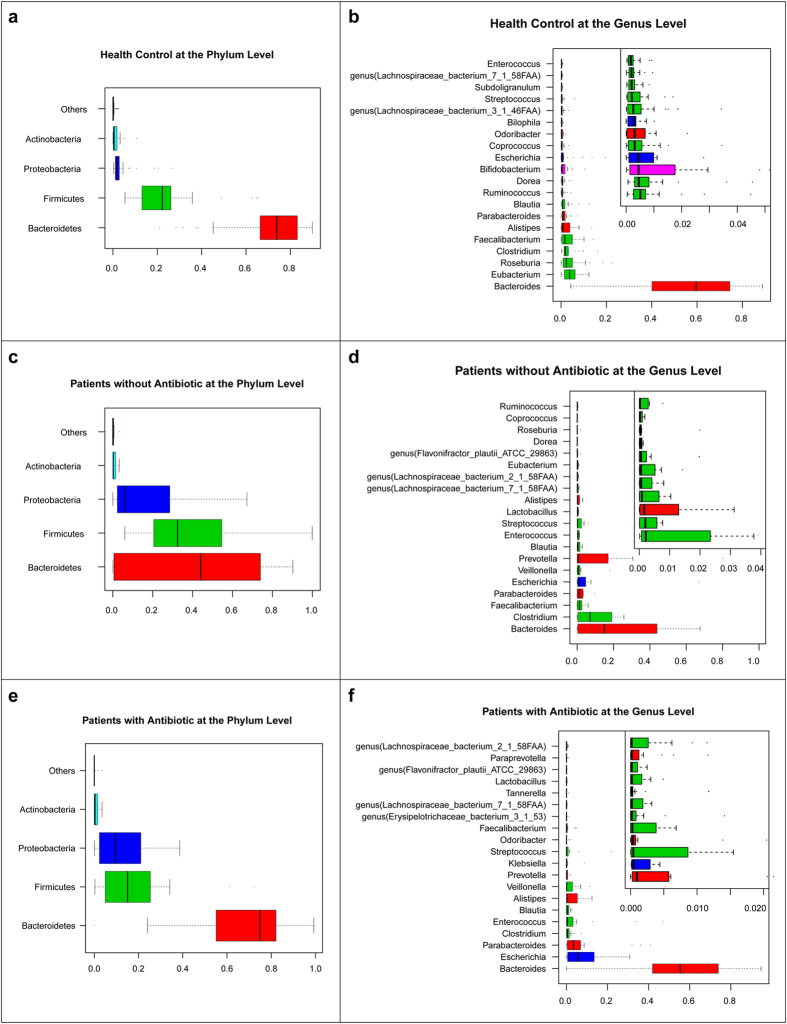
Human gut microbiome abundances and phylogenetic profiles at the phylum and genus levels for healthy controls, H7N9 infected patient with antibiotics and without antibiotics. (**a**) A phylum abundance variation box plot for the top abundant phylum in the healthy controls is shown. The boxes represent the inter quartile range (IQR), from the first and third quartiles, and the inside line represents the median. The whiskers denote the lowest and highest values within 1.53 IQR from the first and third quartiles. The circles represent outliers beyond the whiskers. (**b**) The 20 most abundant genera in the healthy controls are shown. The colour of each genera corresponds with the colour of its respective phylum. (**c**) A phylum abundance variation box plot for the top abundant one in the patients with antibiotics is shown. (**d**) The 20 most abundant genera in the patients with antibiotics are shown. (**e**) A phylum abundance variation box plot for the top abundant phylum in the patients without antibiotics is shown. (**f**) The 20 most abundant genera in the patients without antibiotics are shown.

**Figure 2:**
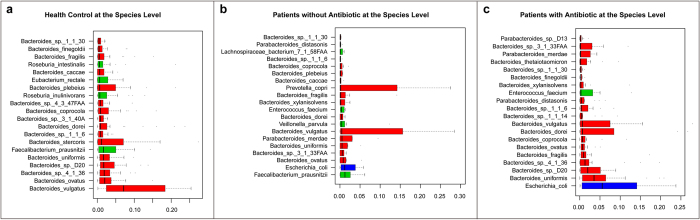
Human gut microbiome abundances and phylogenetic profiles at the species levels for healthy controls, H7N9 infected patient with antibiotics and without antibiotics. (**a**) The 20 most abundant species in the healthy control samples are shown. The colour of each species corresponds with the colour of its respective phylum. (**b**) The 20 most abundant species in the patients with antibiotics samples are shown. (**c**) The 20 most abundant species in the patients without antibiotics samples are shown.

In order to compare gut microbial communities in H7N9 patients with those in healthy individuals, we selected 31 samples from a previous Chinese gut microbiota study matching for age, gender and BMI (Table S2)^10^. Only one sample from each patient (26) and healthy individual (31) was taken for comparison. The clean reads from the sequencing data (Table S3) were aligned against the reference genomes from NCBI and HMP database (Table S4). The relative abundance of phylum, genus and species among healthy control (H) groups, patients treated with antibiotic (AB) and without antibiotic (NAB) are shown in Fig. 1. Phylotypes with a median relative abundance greater than 0.01% (p < 0.01), calculated using the Wilcoxon rank-sum test in either the H group or H7N9 patients were included for comparison. At the phylum level, *Bacteroidetes, Firmicute*s and *Proteobacteria* were dominant in the faecal microbial communities of all groups (Fig. 1a,c,e). Compared with H group, H7N9 patients had fewer *Bacteroidetes* in NAB group and fewer *Firmicutes* in AB group, but higher levels of *Proteobacteria* in both groups. At the genus level, *Bacteroides* was dominant in all groups (Fig. 1b,d,f). In AB group, *Escherichia* and *Parabacteroides* were the second and third genus in faecal microbial communities respectively whereas in NAB group, *Clostridium* and *Parabacteroides* were the second and third. In contrast, in H group *Eubacterium* and *Roseburia* were the second and third, respectively. The top 20 species are shown in Fig. 2. Ofthe top 20 species in AB group and NAB group, *Escherichia coli* represents the most and second most abundant species respectively, indicating that *Escherichia coli* are potentially pathogenic bacteria and whose abundance levels may correlate with the progression of H7N9 infection and associated treatment. We also found *Enterococcus faecium* as enriched in patient groups. It is worthy to note that *Faecalibacterium prausnitzii*, which is recognized as an anti-inflammatory probiotics^17^, was dramatically decreased in AB group. These results show large differences in the microbial communities between the H7N9 patients and the healthy individuals.

**Figure 3:**
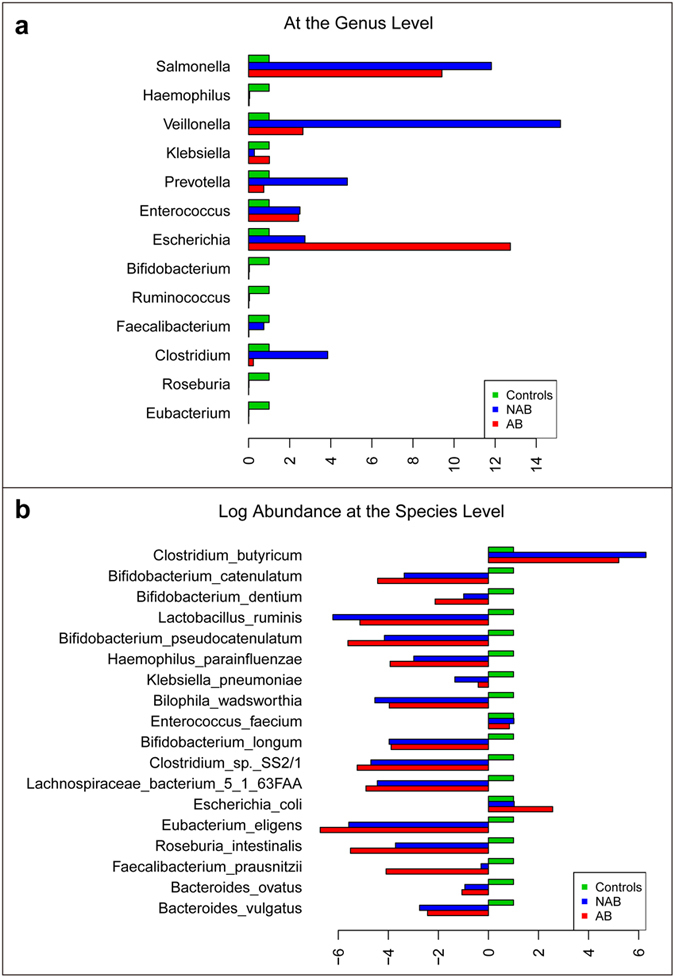
Phylogenetic abundance comparison from selected genus and species and biological diversity comparison among healthy controls, H7N9 infected patient with antibiotics and without antibiotics. (**a**) Phylogenetic median relative abundance comparison from selected genus is shown. The abundance in healthy controls for each genusis set to 1. (**b**) Phylogenetic median relative abundance comparison from selected species is shown. The abundance in healthy controls for each species is set to 1. Log scale is used in X axis, bars on the left showing those species with lower abundance than that in healthy controls.

At the genus level, *Eubacterium*, *Ruminococcus*, *Bifidobacterium* and *Roseburia* dramatically decreased in patient groups compared with controls and a similar trend was seen for *Faecalibacterium* and *Haemophilus* (Fig. 2a). In contrast, in AB and NAB patient groups, the proportion of *Escherichia* were higher than in controls (5.5% versus 1.2%versus 0.4%, respectively) as was *Salmonella*, *Enterococcus* and *Veillonella* (Fig. 3a). At the species level, *Escherichia coli*, the most common species, was significantly more abundant in AB group than in NAB group and H group (5.5% versus 1.2%versus 0.4%, respectively). *Enterococcus faecium* and *Veillonella parvula* were more abundant in the patient groups as was *Clostridium butyricum*, likely because of its administration as probiotic. In contrast, *Bacteroides vulgates* was observed to be the most abundant in H group, significantly more than in AB and NAB groups (Figs 2a and 3b). In addition, we also identified adramatical decline of species such as *Bacteroidesovatus*, *Faecalibacterium prausnitzii*, *Roseburia intestinalis*, *Eubacteriumeligens*, *Bifidobacterium longum* and also *Haemophilus parainfluenzae* in patient groups.

**Figure 4:**
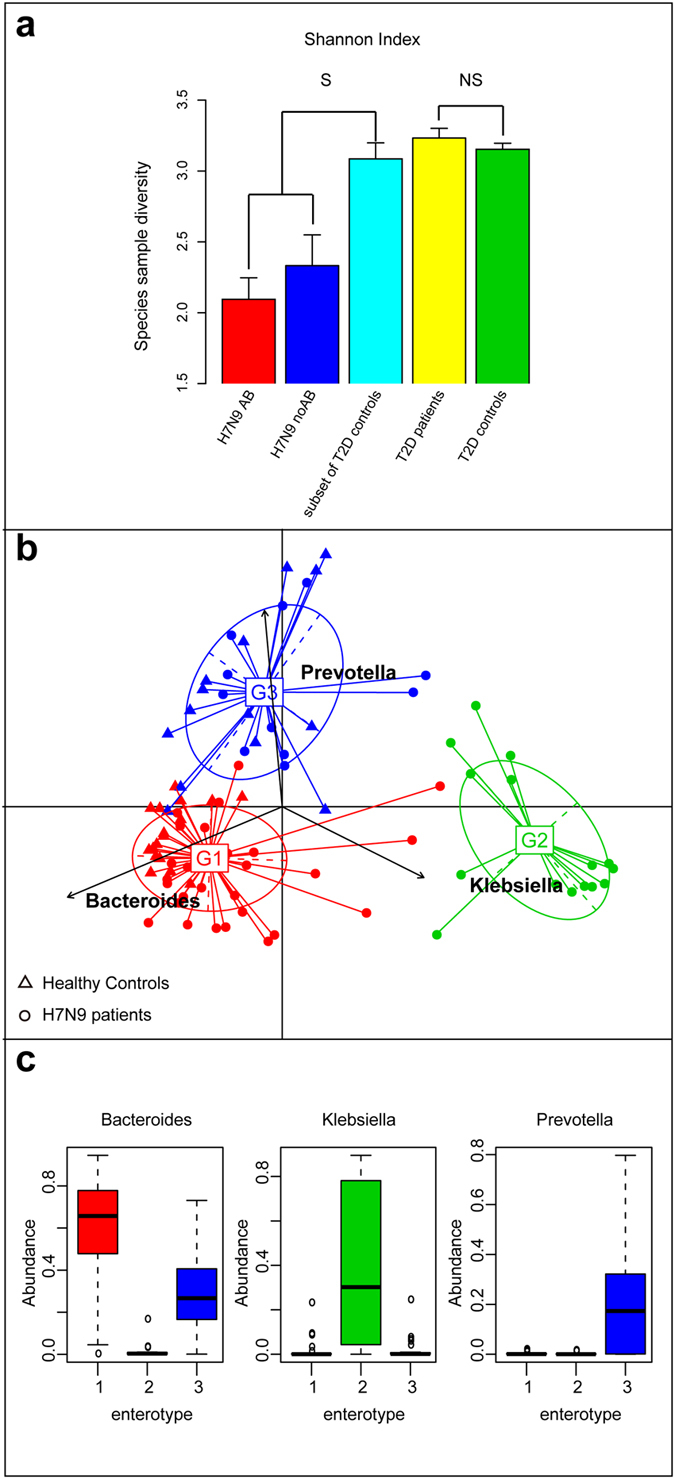
Biological diversity comparison and enterotypes in healthy controls and all patient samples. (**a**) The within-sample diversity (shannonn index) was compared among healthy controls, H7N9 infected patients with antibiotics and without antibiotics; also between T2D patients and controls. The bars denote the within-sample diversity in each group. S, significant; NS, not significant; and the error bar denote standard error. (**b**) PCA result of three enterotypes in genus level is shown. The clustering of the genus and species compositions of all H7N9 infected patient and healthy controls by mapping the metagenome reads to the reference genome sequences using an 85% similarity threshold. (**c**) Abundances of the main contributors of each enterotype are shown.The coloured boxes represent the inter quartile range (IQR), from the first and third quartiles, and the line inside represents the median. The circles represent the outliers beyond the whiskers.

Effects of influenza virus infection on gut microbial communities have not been previously reported. In contrast, it is known that antibiotic administration has an immediate effect, with reducing microbiota diversity^11^. Here we compared the within-sample diversity (Shannon index) among healthy controls and H7N9 infected patients treated with (n = 17) or without (n = 9) antibiotics (Fig. 4a). Very interestingly, viral infection led to a significant decrease of diversity, there was significant difference in the within-sample diversity between NAB group and control (p < 0.01, Student t-test), also between AB group and control (p < 0.001, Student t-test). The decreased diversity is further exacerbated by antibiotics administration, although the difference was not statistically significant between AB and NAB group (p = 0.26 by the Student t-test).

To assess the effects of treatments on gut microbial communities, we selected patients from whom at least one sample was obtained for >4 days of treatment (n = 7; Table S5). It has been shown previously that antibiotic perturbation may shift the gut microbiota community structure to an alternatives state^12^. We examined the enterotypes of all samples (n = 93) from H7N9 virus infected patients and controls (n = 31) by PAM clustering with four distance metrics method (Fig. S1). The best cluster number at the genus level was three (Fig. S1). Two enterotypes were driven by the genera *Bacteroides* and *Prevotella* similar to previous studies in European and Chinese cohorts (Fig. 4c)^9^,^10^. The third enterotype was driven by the genus *Klebsiella* and includes only the H7N9 infected patients. We hypothesize that the infection causes the shift of the communities to this enterotype, which is different from those previously reported. Interestingly, the samples from same patient fluctuate between different enterotypes within a short time (<8 days; Table S3). Furthermore, assessment of the proximity of patients by the Spearman correlation analysis indicates that different samples from the same patient do not cluster together (Fig. S2). We conclude that the gut microbial communities in patients are unstable whereas in the healthy individuals they are rather stable^18^. This may be due, at least in part, to the very significant loss of diversity observed upon infection.

**Figure 5:**
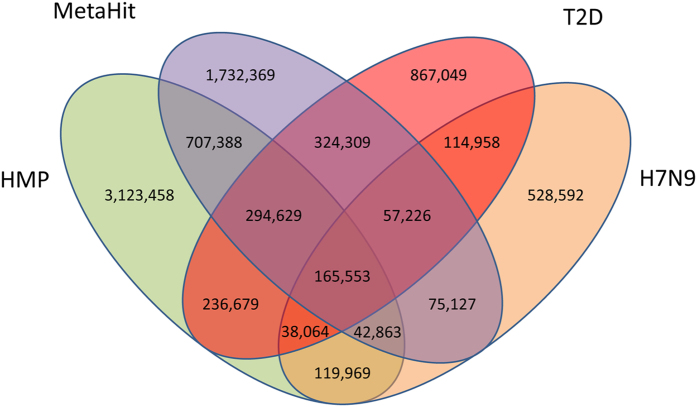
Venn diagram showing the overlap of the current major human microbiome gene set. The total gene number in each gene set and the overlapping areas are listed. (LC: liver cirrhosis gene set, T2D: type 2 diabetes gene set, MetaHIT: MetaHIT gene set, HMP gut: HMP gut gene set).

**Figure 6:**
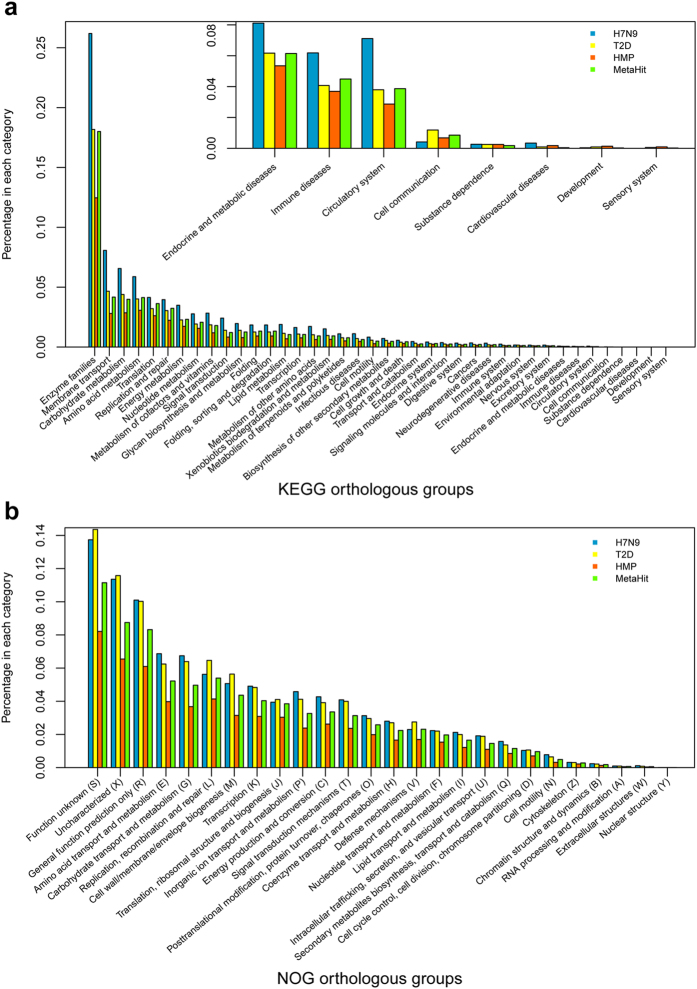
The distribution of the eggNOG and KEGG functional categories for gene markers that shared by all four gene sets in LC, T2D, MetaHIT or HMP gut gene set. (**a**) A comparison of the eggNOG categories for the enriched gene markers is shown by percentage. (**b**) A comparison of the KEGG pathway categories for the enriched gene markers is shown by percentage.

A H7N9 infected patient gut microbial gene set was constructed using MetaGene by removing the redundant ORFs using pair-wise alignment comparison as having 95% identity over 90% of the shorter ORF length^19^. Totally 3,592,885 open reading frames (ORFs) from the 1.3 million contigs were predicted. The final non-redundant gut gene set contained 1,171,674 ORFs with an average length of 722 bp. Then the H7N9 virus infected patient gut microbial gene set was compared to other available gene set like MetaHIT^10^, HMP gut^8^, and T2D^10^. The HMP data set contains sequencing data from multiple human body sites, we here only use gut data from HMP data set. Among all four gene sets, the virus, MetaHIT, HMP gut and T2D gene sets contain 528,592, 1,732,369, 3,123,458 and 867,049 unique genes, respectively (Fig. 5). MetaHIT and HMP gut have 20.55% and 37.06% unique genes, respectively, which imply that the gut genes are very different across continents. Virus and T2D sets contained 6.27% and 10.29% unique genes, respectively, and large differences were observed in the two gene sets derived from Chinese cohorts. We next investigate the functional role of the shared or uniquely present genes. We annotated the unique genes in every gene set into KEGG database, because there is huge gene number difference among four gene sets, so percentage of genes in each gene set is used for comparison. The most abundant KEGG orthologous group in all four gene sets was enzyme families, and then membrane transport and Carbohydrate metabolism (Fig. 6a). We also performed similar analysis using eggNOG database and find the most unique genes function are either function unknown or uncharacterized, the next most abundant categories are general function prediction only, amino acid transport and metabolism (Fig. 6b).

**Figure 7:**
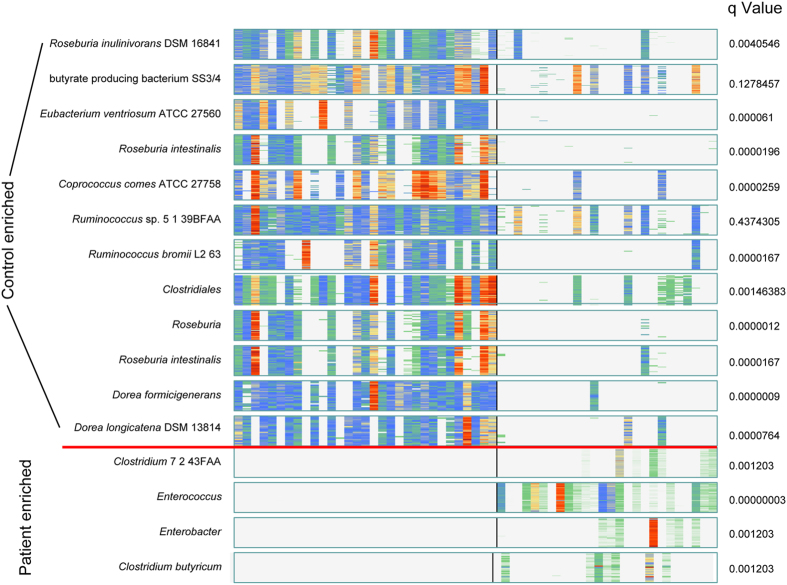
Barcodes of clusters in patient or control group sorted by discriminant significance. Each “barcode” of the clusters where colors from light blue to dark red indicate abundance from low to high respectively, while white is missing. Each line is a gene, each column is an individual who are ordered by group (left is healthy and right is patient) A healthy enriched B case enriched.

To better reveal the enriched species in virus infected patients, MetaGenomic Species(MGS) were constructed^20^. Using the taxonomic characterisation of these MGSs, A number of 13 known MGSs from healthy control associated gene markers and 4 known MGSs from H7N9 virus infected patients associated gene markers were identified (Fig. 7, Table S6). We found that most of the MGS enriched in the control samples were *Roseburia inulinivorans* DSM 16841, butyrate producing bacteriumSS3/4, *Eubacterium ventriosum* ATCC 27560, *Roseburia intestinalis* and *Ruminococcus*. In contrast, most of MGS enriched in the H7N9 patients were *Clostridium* sp. 7 2 43FAA, *Enterococcu*s, *Enterobacter* and *Clostridium butyricum*. It is worthy of note that *Enterococcu*s and *Enterobacter* have been reported to play crucial roles of the gastrointestinal tract infections, lower respiratory tract infections, skin and soft-tissue infections, urinary tract infections, endocarditis, CNS infections, septic arthritis, intra-abdominal infections, and ophthalmic infections^21^,^22^.

## Discussion

In this study, the next generation sequencing technology was applied to analyse, for the first time, the gut microbiota of the H7N9 virus infected patients. For community structure, the changes at the phylum genus and species-level were observed that caused by the H7N9 infection and associated treatment. Interestingly, *Escherichia coli* and *Enterococcus faecium* could be inferred, on the basis of relationship patterns, to be harmful, in line with previous studies, which concluded that they may cause or underlie bacteraemia and intra-abdominal infections^23^,^24^. Interestingly, *Clostridium butyricum* was enriched in both AB and NAB patient groups, furthermore, NAB group has a higher abundance than AB group, indicating that *Clostridium* tablets may exert their functions in the gut (Fig. 3b). In contrast, *Bacteroides vulgatus*, *Bacteroidesovatus*, *Faecalibacterium prausnitzii*, *Roseburia intestinalis*, *Eubacteriumeligens*, *Bifidobacterium longum*and *Haemophilus parainfluenzae* may have a beneficial impact in this specific biological context. In particular, the finding here that *Haemophilus parainfluenzae* was enriched in healthy controls is consistent with the result from MGWAS study^10^, which imply an unknown function of this known pathogen. In our study, patients with H7N9 of different treatment regimens had similar gut community changes. These results led to a conclusion that composition changes observed between H7N9 patients and healthy controls were mainly due to viral infection and associated treatment.

The within sample diversity of gut microbial communities was found to decrease in patients, treated or not with the antibiotics. However, the treatment which included antivirals, probiotics and antibiotics, appeared to help increase the diversity of the gut microbial communities. The clustering analysis of these samples showed that they also could be grouped into three enterotypes as in previous studies but with a different third enterotype driven by *Klebsiella*, which included the patients only. We suggest that this enterotype may correspond to specific microbial communities that form in H7N9 infected individuals. The enterotypes are not stable, possibly due to the conjunction of viral infection and low diversity. The MGS analysis was performed in this study and this was reported possibly to be markers for specific disease^20^. We also found that many MGSs enriched in healthy controls or patients and most of them are consistent with the finding in genus or species abundances changes in this study, such as MGS *Clostridium butyricum* and *Roseburia intestinalis*.

There is also some limitations in this study. First, not all the H7N9 patients in our hospital were enrolled. Second, we were not able to collect the fecal samples from patients in the early stage of infection, because at the time that most of patients were not admitted or transferred to our hospital. Finally, our study didn’t get the stool samples from patients after they discharged from hospital. Further collection is planned in order to assess long-term effects of the infection and the associated treatment in gut microbiota.

## Materials and Methods

### Patient information and sampling

We describe patients who were hospitalized in the First Affiliated Hospital of Zhejiang University for H7N9 virus infection, as confirmed by a real-time RT-PCR.

Patients with confirmed H7N9 infection were interviewed by staff according to a standardized questionnaire to obtain clinical and epidemiologic information, and medical records were reviewed. All participants provided written informed consent for collection and testing of fecal sample prior to entering the study. Research protocols conformed to the ethical guidelines of the 1975Declaration of Helsinki and were approved and monitored by the Institutional Review Board of the First Affiliated Hospital of Zhejiang University.

The fresh fecal samples were collected by nurses from the H7N9 infected patient. Each fresh sample was delivered immediately from the ward to lab in a larger cooler with ice packs where it was divided into aliquots of 200 mg and was immediately stored at −80 °C until next step.

### DNA extraction, library construction and sequencing

A frozen aliquot (200 mg) of each fecal sample was performed by Qiagen QIAamp DNA Stool mini kit according to manufacturer’s introduction. DNA concentration was measured by nanodrop (Thermo Scientific) and its molecular size was estimated by agarose gel electrophoresis. Then DNA libraries were constructed using Illumina TruSeq DNA Sample Prep Kit according to the manufacturer’s instruction. Illumina TruSeq PE Cluster and SBS Kit were used to perform cluster generation, template hybridization, isothermal amplification, linearization, blocking and denaturization and hybridization of the sequencing primers. Paired-end sequencing 2 × 100 bp was performed to sequence all libraries. The base-calling pipeline (Casava 1.8.2 with parameters—use-bases-mask y100n, I6n, Y100n, –mismatches 1, –adapter-sequence) was used to process the raw fluorescent images and call sequences. The insert size inferred by Agilent 2100 was used for all libraries (ranging from 275 to 450).

### Quality control of reads

Reads that mapable to human genome together with their mated reads were removed from each sample using BWA with parameters −n 0.2^25^. Then quality control was preceded with following criteria: a. Reads contained more than 3 N bases were removed, b. Reads contained more than 50 bases with low quality (Q2) were removed, c. Reads contained no more than 10 bases with low quality (Q2) or N base in the tail of reads were trimmed. Resulting filtered reads were considered for alignment analysis.

### Reference genomes set collection, abundance profiling and enterotyping

The reference Bacteria and Archaea genomes genomes were downloaded from NCBI database (version 20120810), including draft genomes, bacterial genomes from HMP (version 2012.6) were also downloaded and intergreted. Soap align 2.21 was used to align paired-end clean reads against reference genomes with parameters –r 2 –m 200 –x 1000. Reads with alignments on same reference genomes were assigned according to the following rules:

A, reads aligned to only one genome, these reads were denoted as unique reads. B, reads aligned to more than one genome, if these genomes come from one species, we denote these reads as unique reads. If the genomes are from more than one species, we denote these reads as multiple reads.

For species *S*, if its abundance is *Ab(S)*, it might have alignments with unique reads set *U* and multi-position match reads *M*, the computation of *Ab(S)* is as follows.


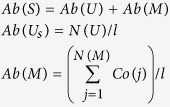


*Ab(U)* and *Ab(M)* mean abundance of uniquely match reads (*U*) and multi-position match reads *(M)* of species *S*, respectively, *N(U)* is the number of uniquely match reads, *N(M)* is the number of multi-position match reads, *l* is length of relative genome.

For each multi-position match read *j*, which has alignments with *Nj* different species, there is a species coefficient *Co(j)* that was calculated as follows.


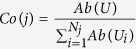


Profiling table at genus level was generated by adding abundance of species into its genera or phylum. For some species that do not have genera, they are denoted as NULL. Based on species profiling and genus profiling table, each sample was assigned enterotype clusters by the three distance metrics: Jensen-Shannon divergence (JSD), Root Jensen-Shannon divergence (rJSD), Bray-Curtis (BC)method using partitioning around medoids Clustering introduced by Arumugam, M. *et al.* and Koren *et al.*^9^,^26^.

### Within sample diversity

Based on the species profile, we calculated the within sample (alpha) diversity to estimate the species richness of a sample. Shannon index was used for each sample with the following formula


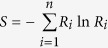


where S is Shannon index, n is number of species found in each sample and R is relative abundance of each species.

### Gene set construction

MetaGeneMark (prokaryotic GeneMark.hmm version 2.8) was used to predict ORFs in assembled proceeded scaffolds (without ambiguous bases). The non-redundant virus infected patient gut gene set was built by pair-wise comparison of all the predicted ORFs using blat and the redundant ORFs were removed using a criterion of 95% identity over 90% of the shorter ORF length, which is consisted with the criterion used in the MetaHIT. We use MetageneMark to predict genes in assembled contigs originally from MetaHIT, T2D and HMP study and performed comparison study using the same method described above.

### Gene markers selection

Gene markers are selected as differentially abundance between the patient and the healthy groups. Wilcoxon tests were employed to compute the probabilities that frequency profiles are not different between the patient and the healthy groups by chance. Then we used Benjamini Hochberg as multiple tests to adjust it. As a result, we got 24,113 and 12,781 gene markers separately based on 0.001 and 0.05 *p* value cutoff in patient and healthy control groups.

### Gene clustering

We clustered the selected gene markers profiles based on above method which have a similar abundance in one individual but different abundance in different individuals. First, the spearman correlation coefficients of gene markers were computed among 57 individuals (26 patients and 31 healthy controls). The less spearman rho value, the more similar between the two genes. Then we used the farthest method to put the genes with 90% similarity together. To validate it, clusters are taxonomically annotated by blating each gene in one cluster to NCBI (complete and draft) and HMP database, Lastly we chose the clusters, that contained more than 20 genes, and selected the taxonomical level by requiring that at least 80% of the genes had a best hit to the same phylogenetic group. 12 and 4 MGS were finally obtained in patient and health cohort.

## Additional Information

**How to cite this article**: Qin, N. *et al.* Influence of H7N9 virus infection and associated treatment on human gut microbiota. *Sci. Rep.* **5**, 14771; doi: 10.1038/srep14771 (2015).

## Supplementary Material

Supplementary figure and table legends

Supplementary Figure S1

Supplementary Figure S2

Supplementary Tables

## Figures and Tables

**Table 1:** Patient Characteristics.

Patient No.	Age	Sex	Antibiotic treatment	Antiviral treatment	Probiotics given	TPF-D given	BMI	Outcomes
1	79	M	Piperacillin-sulbactam, imipenem-cilastatin	Oseltamivir	*Clostridium* tablets, *Bacillus* capsules	Yes	27.1	Outpatient
2	55	F	Piperacillin-sulbactam, piperacillin-tazobactam	Oseltamivir	*Clostridium* tablets	No	27.7	Outpatient
3	68	M	Piperacillin-tazobactam, vancomycin, imipenem-cilastatin,	Oseltamivir	*Clostridium* tablets	Yes	23.0	Outpatient
4	38	M	Vancomycin, piperacillin-tazobactam,	Oseltamivir	*Clostridium* tablets	Yes	22.7	Outpatient
5	43	M	None	Oseltamivir	*Clostridium* tablets	No	23.2	Outpatient
6	57	M	None	Oseltamivir	*Clostridium* tablets	No	18.8	Outpatient
7	75	M	Sulbactam-cefoperazone, moxifloxacin, piperacillin-tazobactam	Oseltamivir	*Clostridium* tablets	No	22.7	Outpatient
8	66	M	Piperacillin-tazobactam, imipenem-cilastatin, sulbactam-cefoperazone	Oseltamivir	*Clostridium* tablets	Yes	29.8	Outpatient
9	80	F	Fluconazole, piperacillin-tazobactam,	Oseltamivir	*Clostridium* tablets	No	21.1	Outpatient
10	59	F	None	Oseltamivir	*Clostridium* tablets, *B. Subtilis* and *E. faecium* enteric coated capsules	No	25.4	Outpatient
11	55	M	None	Oseltamivir	*B. Subtilis* and *E. faecium* enteric coated capsules	No	21.3	Outpatient
12	58	M	Piperacillin-sulbactam	Oseltamivir	None	No	23.4	Outpatient
13	65	M	None	Oseltamivir	*Clostridium* tablets	No	22.9	Outpatient
14	63	M	Fluconazole, piperacillin-tazobactam, moxifloxacin	Oseltamivir	*Clostridium* tablets	No	24.2	Outpatient
15	30	M	Piperacillin-tazobactam, moxifloxacin	Oseltamivir	*Clostridium* tablets, *B. Subtilis* and *E. faecium* enteric coated capsules	No	26.0	Outpatient
16	48	M	Moxifloxacin	Oseltamivir	*Clostridium* tablets	No	26.0	Outpatient
17	38	M	Moxifloxacin	Oseltamivir	*Clostridium* tablets	No	23.2	Outpatient
18	33	F	Moxifloxacin	Oseltamivir	*Clostridium* tablets	No	20.8	Outpatient
19	67	M	Moxifloxacin, vancomycin, piperacillin-tazobactam	Oseltamivir	*Clostridium* tablets	Yes	26.0	Died
20	73	F	Moxifloxacin, vancomycin, tigecycline, piperacillin-tazobactam, sulbactam-cefoperazone,	Oseltamivir	*Clostridium* tablets	Yes	25.1	Died
21	75	M	Moxifloxacin, vancomycin, piperacillin-tazobactam, sulbactam- cefoperazone	Oseltamivir	*Clostridium* tablets	Yes	25.3	Outpatient
22	68	F	None	Oseltamivir	*Clostridium* tablets	No	25.7	Outpatient
23	41	M	None	Oseltamivir	*Clostridium* tablets	No	19.4	Outpatient
24	37	F	None	Oseltamivir	*Clostridium* tablets	No	27.3	Outpatient
25	80	M	Levofloxacin, piperacillin-tazobactam	Oseltamivir	*Clostridium* tablets	No	22.6	Outpatient
26	39	M	None	Oseltamivir	Clostridium tablets	No	20.1	Outpatient

TPF-D: Enteral Nutritional Emulsion.

## References

[b1] Chen Y. *et al.* Human infections with the emerging avian influenza A H7N9 virus from wet market poultry: clinical analysis and characterisation of viral genome. Lancet 381, 1916–1925, 10.1016/S0140-6736(13)60903-4 (2013). 23623390 PMC7134567

[b2] Gao R. *et al.* Human infection with a novel avian-origin influenza A (H7N9) virus. New England Journal of Medicine 368, 1888–1897 (2013). 23577628 10.1056/NEJMoa1304459

[b3] Li Q. *et al.* Preliminary report: epidemiology of the avian influenza A (H7N9) outbreak in China. New England Journal of Medicine (2013).

[b4] Liu D. *et al.* Origin and diversity of novel avian influenza A H7N9 viruses causing human infection: phylogenetic, structural, and coalescent analyses. Lancet 381, 1926–1932, 10.1016/S0140-6736(13)60938-1 (2013). 23643111

[b5] Gao H. N. *et al.* Clinical findings in 111 cases of influenza A (H7N9) virus infection. The New England journal of medicine 368, 2277–2285, 10.1056/NEJMoa1305584 (2013). 23697469

[b6] Clemente J. C., Ursell L. K., Parfrey L. W. & Knight R. The impact of the gut microbiota on human health: an integrative view. Cell 148, 1258–1270, 10.1016/j.cell.2012.01.035 (2012). 22424233 PMC5050011

[b7] O’Hara A. M. & Shanahan F. The gut flora as a forgotten organ. EMBO reports 7, 688–693 (2006). 16819463 10.1038/sj.embor.7400731 PMC1500832

[b8] Human Microbiome Project, C. A framework for human microbiome research. Nature 486, 215–221, 10.1038/nature11209 (2012). 22699610 PMC3377744

[b9] Arumugam M. *et al.* Enterotypes of the human gut microbiome. Nature 473, 174–180 (2011). 21508958 10.1038/nature09944 PMC3728647

[b10] Qin J. *et al.* A metagenome-wide association study of gut microbiota in type 2 diabetes. Nature 490, 55–60, 10.1038/nature11450 (2012). 23023125

[b11] Jernberg C., Lofmark S., Edlund C. & Jansson J. K. Long-term ecological impacts of antibiotic administration on the human intestinal microbiota. The ISME journal **1**, 56-66, 10.1038/ismej.2007.3 (2007). 18043614

[b12] Dethlefsen L. & Relman D. A. Incomplete recovery and individualized responses of the human distal gut microbiota to repeated antibiotic perturbation. Proceedings of the National Academy of Sciences of the United States of America 108 **Suppl 1**, 4554–4561, 10.1073/pnas.1000087107 (2011). 20847294 PMC3063582

[b13] Hatakka K. *et al.* Effect of long term consumption of probiotic milk on infections in children attending day care centres: double blind, randomised trial. Bmj 322, 1327 (2001). 11387176 10.1136/bmj.322.7298.1327 PMC32161

[b14] Lin J. S. *et al.* Different effects of probiotic species/strains on infections in preschool children: A double-blind, randomized, controlled study. Vaccine 27, 1073–1079, 10.1016/j.vaccine.2008.11.114 (2009). 19114073

[b15] Ichinohe T. *et al.* Microbiota regulates immune defense against respiratory tract influenza A virus infection. Proceedings of the National Academy of Sciences of the United States of America 108, 5354–5359, 10.1073/pnas.1019378108 (2011). 21402903 PMC3069176

[b16] Leung R. K. *et al.* Modulation of potential respiratory pathogens by pH1N1 viral infection. Clinical microbiology and infection: the official publication of the European Society of Clinical Microbiology and Infectious Diseases 19, 930–935, 10.1111/1469-0691.12054 (2013). 23167452

[b17] Sokol H. *et al.* Faecalibacterium prausnitzii is an anti-inflammatory commensal bacterium identified by gut microbiota analysis of Crohn disease patients. Proceedings of the National Academy of Sciences 105, 16731–16736 (2008). 10.1073/pnas.0804812105 PMC2575488 18936492

[b18] Turnbaugh P. J. *et al.* A core gut microbiome in obese and lean twins. Nature 457, 480–484, 10.1038/nature07540 (2009). PMC2677729 19043404

[b19] Noguchi H., Park J. & Takagi T. MetaGene: prokaryotic gene finding from environmental genome shotgun sequences. Nucleic acids research 34, 5623–5630, 10.1093/nar/gkl723 (2006). 17028096 PMC1636498

[b20] Le Chatelier E. *et al.* Richness of human gut microbiome correlates with metabolic markers. Nature 500, 541–546 (2013). 23985870 10.1038/nature12506

[b21] Arias C. A. & Murray B. E. The rise of the Enterococcus: beyond vancomycin resistance. Nature reviews. Microbiology 10, 266–278, 10.1038/nrmicro2761 (2012). 22421879 PMC3621121

[b22] Sanders W. & Sanders C. C. Enterobacter spp.: pathogens poised to flourish at the turn of the century. Clinical Microbiology Reviews 10, 220–241 (1997). 9105752 10.1128/cmr.10.2.220 PMC172917

[b23] McClean K., Sheehan G. & Harding G. Intraabdominal infection: a review. Clinical Infectious Diseases 19, 100–116 (1994). 7948510 10.1093/clinids/19.1.100

[b24] Pinholt M. *et al.* Incidence, clinical characteristics and 30‐day mortality of enterococcal bacteraemia in Denmark 2006–2009: a population‐based cohort study. Clinical Microbiology and Infection 20, 145–151 (2014). 23647880 10.1111/1469-0691.12236

[b25] Li H. & Durbin R. Fast and accurate short read alignment with Burrows-Wheeler transform. Bioinformatics 25, 1754–1760, 10.1093/bioinformatics/btp324 (2009). 19451168 PMC2705234

[b26] Koren O. *et al.* A guide to enterotypes across the human body: meta-analysis of microbial community structures in human microbiome datasets. PLoS computational biology 9, e1002863, 10.1371/journal.pcbi.1002863 (2013). 23326225 PMC3542080

