# Supplementary material for: Influence of H7N9 virus infection and associated treatment on human gut microbiota
Source: Sci Rep. 2015 Oct 22;5:14771. doi: 10.1038/srep14771 (PMC4614822; doi:10.1038/srep14771)
Supplement: Supplementary figure and table legends [file srep14771-s1.pdf]

Supplementary information

Influence of H7N9 virus infection and associated treatment on human gut microbiota

Running title: H7N9 influence on human gut microbiota

Nan Qin[1,2]*†, Beiwen Zheng[1,2]*, Jian Yao[1,2]*, Lihua Guo[1], Jian Zuo[1], Lingjiao Wu[1], Jiawei Zhou[1,2], Lin Liu[1,2], Jing Guo[1], Shujun Ni[1], Ang Li[1], Yixin Zhu[1,2], Weifeng Liang[1,2], Yonghong Xiao[1,2], S. Dusko Ehrlich[3,4], LanJuan Li[1,2]†

[1]State Key Laboratory for Diagnosis and Treatment of Infectious Diseases, the First Affiliated College of Medicine, Zhejiang University, 310003 Hangzhou,China; [2]Collaborative Innovation Center for Diagnosis and Treatment of Infectious Diseases, Zhejiang University, 310003 Hangzhou, China and [3]Metagenopolis, Institut National de la Recherche Agronomique, 78350, Jouy en Josas, France; [4]King's College London, Centre for Host-Microbiome Interactions, Dental Institute Central Office, Guy's Hospital, London Bridge, London SE1 9RT, UK.

*These authors contributed equally to this work.

†Correspondence: Prof. Lanjuan Li, State Key Laboratory for Diagnosis and Treatment of Infectious Diseases, First Affiliated Hospital, College of Medicine, Zhejiang University, No. 79 Qingchun Rd., Hangzhou City 310003, China (ljli@zju.edu.cn). or Dr. Nan Qin, State Key Laboratory for Diagnosis and Treatment of Infectious Diseases, First Affiliated Hospital, College of Medicine, Zhejiang University, No. 79 Qingchun Rd., Hangzhou City 310003, China (nqin@zju.edu.cn).

SUPPLEMENTARY FIGURE AND TABLE LEGENDS

Figure S1: Clustering scores for enterotypes at the genus levelusing four different methods: Prediction strength scores, Calinski-Harabasz, average silhouette scores and WB score. Each one has three different metrics. The x-axis shows the cluster number,and the y-axis shows the score.

Figure S2：Clustering results based on the Spearman correlation co efficiency of all samples. A: At the species level. B: At the genus level.

Table S1: Patients characteristics.

Table S2: Phenotype information of 31 healthy controls.

Table S3: Data Production of 93 samples from 26 H7N9 virus infected patients.

Table S4: Reference genomes downloaded from the NCBI and HMP database (version 20120810)

Table S5: Shannon index of the samples from the selected patients.

Table S6: MGS annotation.
