# Supplementary material for: Influence of H7N9 virus infection and associated treatment on human gut microbiota
Source: Sci Rep. 2015 Oct 22;5:14771. doi: 10.1038/srep14771 (PMC4614822; doi:10.1038/srep14771)
Supplement: Supplementary Figure S2 [file srep14771-s3.pdf]

Supplementary information

Influence of H7N9 virus infection and associated treatment on human gut microbiota

Running title: H7N9 influence on human gut microbiota

Nan Qin[1,2]*†, Beiwen Zheng[1,2]*, Jian Yao[1,2]*, Lihua Guo[1], Jian Zuo[1], Lingjiao Wu[1], Jiawei Zhou[1,2], Lin Liu[1,2], Jing Guo[1], Shujun Ni[1], Ang Li[1], Yixin Zhu[1,2], Weifeng Liang[1,2], Yonghong Xiao[1,2], S. Dusko Ehrlich[3,4], LanJuan Li[1,2]†

[1]State Key Laboratory for Diagnosis and Treatment of Infectious Diseases, the First Affiliated College of Medicine, Zhejiang University, 310003 Hangzhou,China; [2]Collaborative Innovation Center for Diagnosis and Treatment of Infectious Diseases, Zhejiang University, 310003 Hangzhou, China and [3]Metagenopolis, Institut National de la Recherche Agronomique, 78350, Jouy en Josas, France; [4]King's College London, Centre for Host-Microbiome Interactions, Dental Institute Central Office, Guy's Hospital, London Bridge, London SE1 9RT, UK.

*These authors contributed equally to this work.

†Correspondence: Prof. Lanjuan Li, State Key Laboratory for Diagnosis and Treatment of Infectious Diseases, First Affiliated Hospital, College of Medicine, Zhejiang University, No. 79 Qingchun Rd., Hangzhou City 310003, China (ljli@zju.edu.cn). or Dr. Nan Qin, State Key Laboratory for Diagnosis and Treatment of Infectious Diseases, First Affiliated Hospital, College of Medicine, Zhejiang University, No. 79 Qingchun Rd., Hangzhou City 310003, China (nqin@zju.edu.cn).

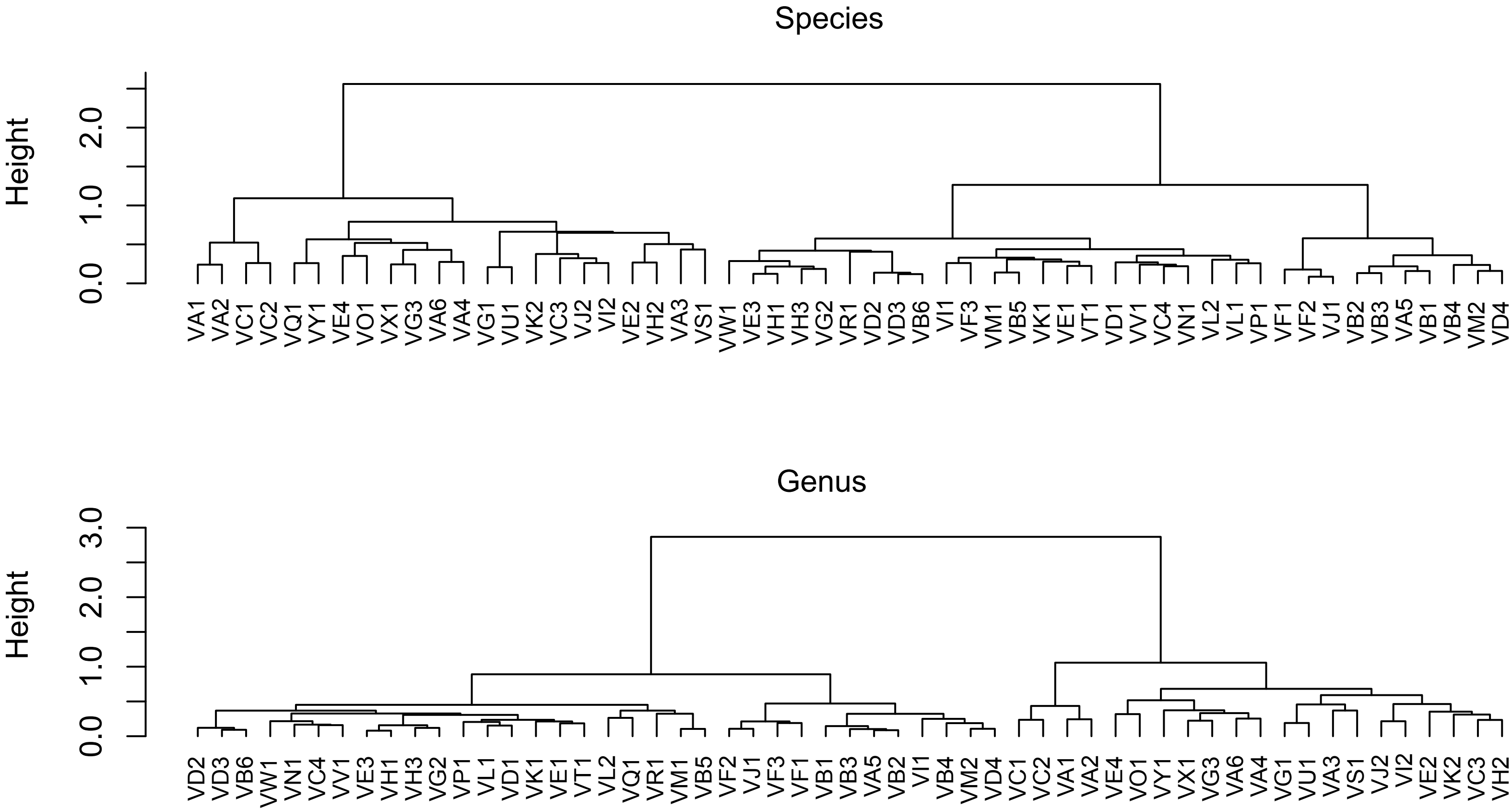

Species
Height
0.0
1.0
2.0
VA1 VA2 VC1 VC2 VQ1 VY1 VE4 VO1 VX1 VG3 VA6 VA4 VG1 VU1 VK2 VC3 VJ2 VI2 VE2 VH2 VA3 VS1 VW1 VE3 VH1 VH3 VG2 VR1 VD2 VD3 VB6 VI1 VF3 VM1 VB5 VK1 VE1 VT1 VD1 VV1 VC4 VN1 VL2 VL1 VP1 VF1 VF2 VJ1 VB2 VB3 VA5 VB1 VB4 VM2 VD4
Genus
Height
0.0
1.0
2.0
3.0
VD2 VD3 VB6 VW1 VN1 VC4 VV1 VE3 VH1 VH3 VG2 VP1 VL1 VD1 VK1 VE1 VT1 VL2 VQ1 VR1 VM1 VB5 VF2 VJ1 VF3 VF1 VB1 VB3 VA5 VB2 VI1 VB4 VM2 VD4 VC1 VC2 VA1 VA2 VE4 VO1 VY1 VX1 VG3 VA6 VA4 VG1 VU1 VA3 VS1 VJ2 VI2 VE2 VK2 VC3 VH2
